# Pressure induced elastic softening in framework aluminosilicate- albite (NaAlSi_3_O_8_)

**DOI:** 10.1038/srep34815

**Published:** 2016-10-13

**Authors:** Mainak Mookherjee, David Mainprice, Ketan Maheshwari, Olle Heinonen, Dhenu Patel, Anant Hariharan

**Affiliations:** 1 Earth Materials Laboratory, Earth, Ocean and Atmospheric Sciences, Florida State University, Tallahassee, FL, 32310, USA; 2 Géosciences Montpellier UMR CNRS 5243, Université Montpellier, 34095, Montpellier, France; 3 Center for Simulation and Modeling, University of Pittsburgh, Pittsburgh, PA, 15260, USA; 4 Materials Science Division, Argonne National Laboratory, Argonne, IL 60439, USA; 5 Earth and Atmospheric Sciences, Cornell University, NY 14850, USA

## Abstract

Albite (NaAlSi_3_O_8_) is an aluminosilicate mineral. Its crystal structure consists of 3-D framework of Al and Si tetrahedral units. We have used Density Functional Theory to investigate the high-pressure behavior of the crystal structure and how it affects the elasticity of albite. Our results indicate elastic softening between 6–8 GPa. This is observed in all the individual elastic stiffness components. Our analysis indicates that the softening is due to the response of the three-dimensional tetrahedral framework, in particular by the pressure dependent changes in the tetrahedral tilts. At pressure <6 GPa, the PAW-GGA can be described by a Birch-Murnaghan equation of state with 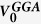 = 687.4 Å^3^, 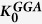 = 51.7 GPa, and 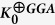 = 4.7. The shear modulus and its pressure derivative are 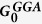 = 33.7 GPa, and 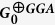 = 2.9. At 1 bar, the azimuthal compressional and shear wave anisotropy 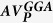 = 42.8%, and 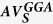 = 50.1%. We also investigate the densification of albite to a mixture of jadeite and quartz. The transformation is likely to cause a discontinuity in density, compressional, and shear wave velocity across the crust and mantle. This could partially account for the Mohorovicic discontinuity in thickened continental crustal regions.

Plagioclase feldspar is one of the most important mineral solid-solution series between the end-members- albite (NaAlSi_3_O_8_) and anorthite (CaAl_2_Si_2_O_8_). Plagioclase is ubiquitous in crustal rocks in the Earth and terrestrial planetary bodies, including Moon^1^. Sodic-plagiocase or albite is a major constituent of several rock types includinggranite, granodiorite, diorite, tonalite, and basalt. The modal abundances of plagioclase in these rocks vary between 16 and 60% 2. In hydrothermal ‘Kluftalbite’ veins the modal abundance of albite rich plagioclase could be nearly 100% 3. In subducted oceanic crust, albite is widely distributed in metamorphosed mid-ocean ridge basalt (MORB) i.e., zeolite to garnet granulite facies rocks with a modal abundance ranging between ~18–22% 4. Albitization is a common metasomatic process in granitic and mafic lithologies transforming Ca-bearing plagioclase to almost pure albite^5^. Albite is also abundant in unconsolidated sediments, either as detrital grains of various compositions or grows as authigenic crystals of pure albite^6^, and has been reported in oil shales associated with organic matter^7^.

Albite also occurs in wide variety of planetary materials including chondrites ~10% and Martian meteorites^8^,^9^. Sodic-plagioclase has also been identified in the exposed rocks and soils at several the landing sites of the Mars Exploration Rovers^10^ and as a widespread component of andesitic lava flows on the surface^11^. In addition, the Thermal Emission Spectrometer on the Global Surveyor spacecraft orbiting Mars have also identified sodic-plagicoclase as dust particles in the atmosphere^12^. There is also speculation that albite may be present on Mercury^13^,^14^.

To understand the dynamics of the planetary crust it is important to gain constraints on the physical properties of planetary crusts and its constituent rocks and minerals. Physical properties of plagioclase feldspar vary strongly as a function of chemistry between the albite and anorthite end-members. For instance, the elastic parameter such as bulk modulus increases by 60% from albite to anorthite^15^ whereas the transport properties such as viscosity vary over three orders of magnitude at constant temperature^16^. It is fundamental to address how the crystal structure and chemistry influences the physical property across the plagioclase solid solution. Albite also forms a solid solution with orthoclase (KAlSi_3_O_8_) i.e., the alkali feldspar solid solution series [(Na,K)AlSi_3_O_8_]. Albite is therefore the key model crystal structure for the aluminosilicate feldspar group of mineral. The crystal structure of albite consists of linked corner-sharing TO_4_ tetrahedral units (where T = Al, Si) forming a three- dimensional aluminosilicate framework, often referred to as *crankshaft* structure.

Despite being a major component in continental and oceanic crust and occurring in wide variety of Earth and planetary settings, our knowledge of fundamental physical properties, *e.g.* elasticity, of albite is rather limited. This is primarily owing to its complex crystal structure and triclinic space group symmetry. In a pioneering experimental study, ultrasonic velocities were measured for plagioclase single-crystals with 9–60 mol % of anorthite. Ultrasonic measurements at 1 bar and 298 K, in adequate directions of the single-crystals allowed for the determination of 13 independent elastic constants *i.e.*, the elastic stiffness tensor with monoclinic rather than the true triclinic symmetry were determined^17^. The reported values were later revised, but still used monoclinic symmetry^18^. More recently, the full elastic tensor for albite^19^, plagioclase feldspars^20^, and alkali feldspars^21^ have been reported at ambient pressure and room temperature. In addition, room pressure elastic constants for plagioclase feldspar have also been predicted using *ab initio* methods^22^. With increasing depth in the crust, i.e., with increasing temperature and pressure, the crystal structure of albite becomes thermodynamically unstable, while denser mineral phases such as jadeite and quartz replace albite^23^. However, the transition to from albite to a mixture of jadeite and quartz also requires elevated temperatures. It is therefore likely that albite may persist as a metastable phase to greater depths^24^. Thus, it is important to know the pressure-dependence of this crystal structure and its physical properties, including elasticity. Several high-pressure studies have therefore been devoted to elucidating the crystal structure and compressibility^24^,^25^,^26^ of albite. These studies have indicated a pressure-induced softening of bulk moduli at around 6 GPa. We still do not have any understanding of how the full elastic stiffness tensor and the bulk and shear moduli change upon compression. In order to understand the elasticity and anisotropy of planetary crusts, we have performed first principles calculations of albite with triclinic symmetry at high-pressure. The equation of state and elastic parameters of albite feldspar at high-pressures will be of crucial importance for the thermodynamic database to predict phase relations and velocity discontinuities across crust and mantle^27^,^28^,^29^,^30^,^31^.

## Results

**Figure 1:**
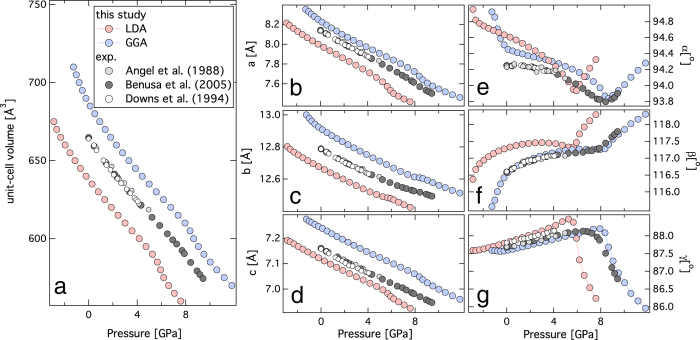
Pressure dependence of unit-cell volume and lattice parameters. (**a**) unit-cell volume, (**b**) lattice parameters *a*, (**c**) *b*, (**d**) *c*-axes, angular lattice parameter (**e**) α, (**f**) β, and (**g**) γ as a function of pressure. ‘Red’ and ‘blue’ symbols represent LDA and GGA results. Experimental results are denoted by- ‘white’ symbols^24^, ‘light grey’ symbols^25^, and ‘dark grey’ symbols^26^.

The compression behavior of the albite could be described with a Birch-Murnaghan equation of state^32^. The unit-cell volume and cell axes predicted with LDA and GGA bracket the previous X-ray diffraction results (Fig. 1). LDA typically overbinds, whereas the GGA under-binds, *i.e*., unit-cell volume data are slightly larger than the experimental results (Fig. 1). The inter-axial angles from our structural optimizations of albite using GGA are in excellent agreement with the previous X-ray diffraction studies^26^ (Fig. 1). The unit-cell volume predicted by GGA at 0 GPa shows 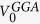 > 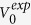 by ~3.4%, whereas unit-cell volume predicted by LDA shows 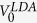 < 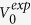 by ~4.0% (Table S1, Fig. 1). The bulk moduli predicted using GGA at 0 GPa shows 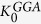 < 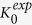 by ~1.1% whereas the bulk moduli predicted using LDA shows 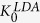 > 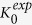 by ~14.5% (Table S1, Fig. 1).

**Figure 2:**
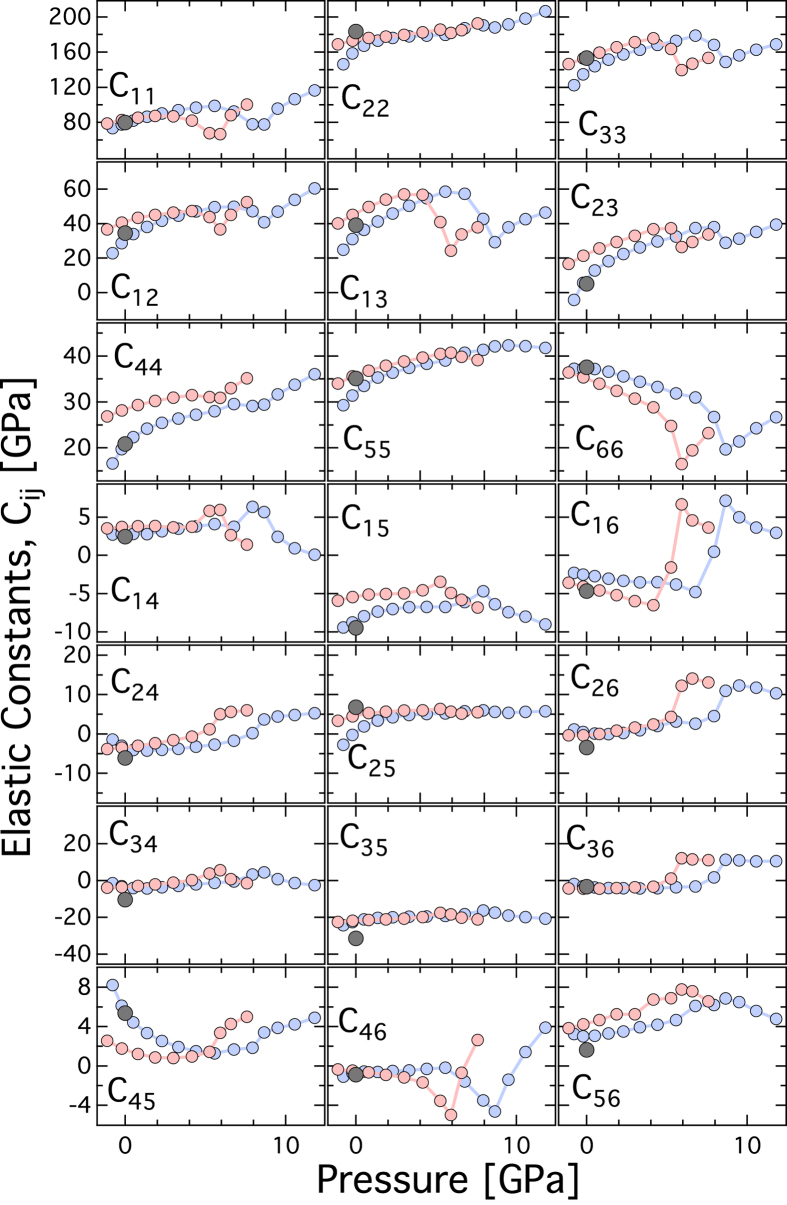
Elastic constants as a function of pressure. (**a**) Plot of the components of the full elastic stiffness tensor vs. pressure. There are three columns and seven rows, a total of 21 graphs, each representing the 21 components of the elastic constants. Each graph is labeled with the corresponding component. ‘Red’ and ‘blue’ symbols represent LDA and GGA results respectively. Experimental results are denoted by- ‘dark grey symbols’^19^. At high-pressures, both LDA and GGA results indicate the anomalous behavior of the elastic stiffness components.

Albite (triclinic symmetry) has 21 non-zero independent elastic constants, *c*_*ij*_ - three diagonal and compressional elastic stiffness components with, *i *= *j*, and *i *= 1–3; three diagonal and shear elastic stiffness components with *i *= *j*, and *i *= 4–6, and 15 off-diagonal elastic stiffness components with *i *≠ *j* and *i *= 1–6 33. The agreement between the predicted elastic constants at room pressures from this study and previous experimental results are quite good^19^ (Fig. 2). For most of the components of full elastic moduli tensor, the experimental result at ambient condition is bracketed by the predictions based on LDA and GGA. The variation of elastic stiffness components with pressure can be explained with a finite-strain formulation^29^,^34^. Typically, the elastic moduli stiffen (*i.e.*, 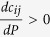) upon compression, however, in the case of albite, most of the predicted elastic moduli based on LDA and GGA exhibit anomalous behavior, (*i.e.*, 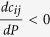) between 4–6 and 6–8 GPa respectively (Fig. 2). The predicted anomalous behavior in the elastic parameters is in good agreement with the single-crystal X-ray diffraction study at high-pressure^26^. The compressional elastic moduli exhibit the relation,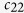 > *c*_33_ > *c*_11_, and the relation persists even at higher pressure. The off-diagonal longitudinal elastic moduli exhibit the relation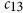 > *c*_12_ > *c*_23_. The diagonal shear components of elastic moduli exhibit the relation *c*_66_ ≥ *c*_55_ > *c*_44_. While diagonal shear components *c*_55_ and *c*_44_ stiffen upon compression, *c*_66_ softens upon compression with 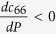. Additional off-diagonal components also exhibit anomalous behavior upon compression (Fig. 2, Table S2a,b). The predicted elastic moduli based on LDA are mostly stiffer than the GGA, *i.e.*, 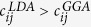.

## Discussions

**Figure 3:**
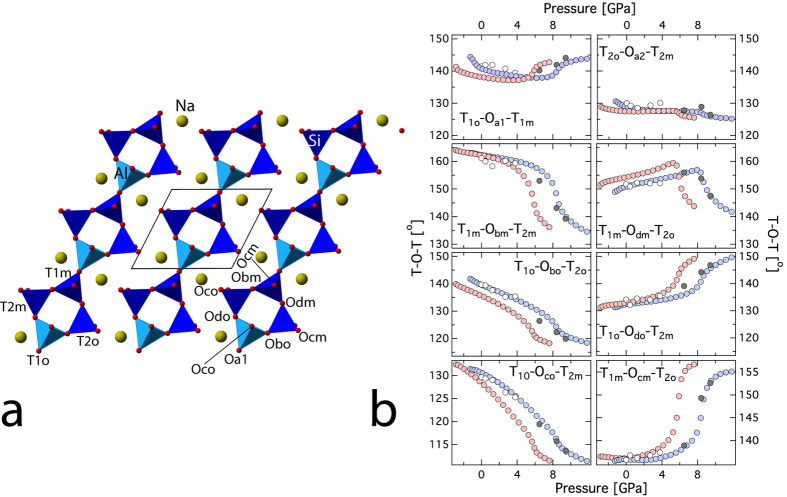
Crystal structure of albite as a function of pressure. (**a**) Representation of the chains of four membered AlO_4_ (light blue) and SiO_4_ (dark blue) tetrahedral units along the [001] showing the T-O-T linkages. Also, shown are the Na atoms (golden sphere) in the slice of albite structure (0.5 < *y* < 0.9). (**b**) Plot of the tetrahedral (TO_4_) framework, i.e., the T-O-T (T = Si, Al) linkage vs. pressure. LDA (light red), GGA (light blue), Experiments: white open symbols^24^ and grey^26^.

**Figure 4:**
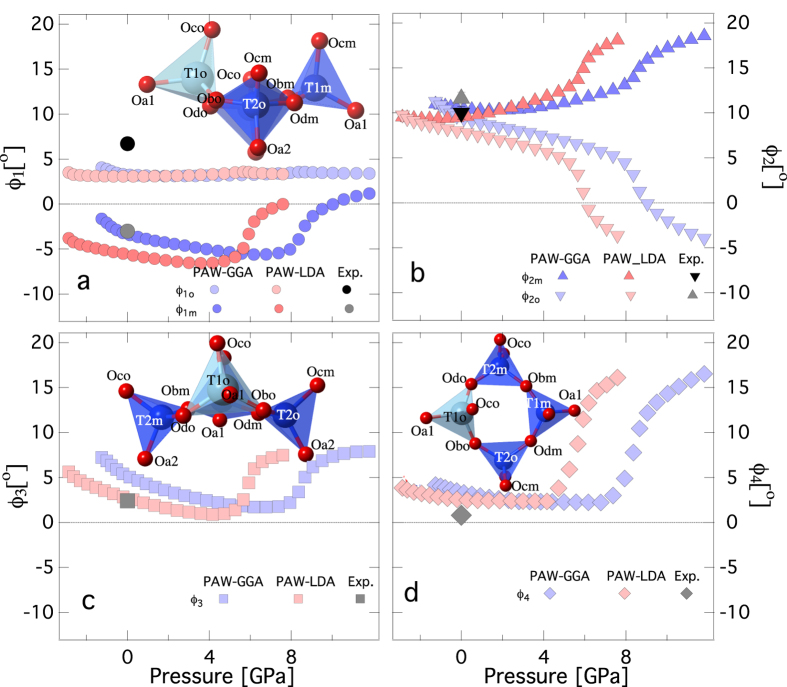
Tetrahedral tilts of albite as a function of pressure. (**a**) for ϕ_1_ (**b**) ϕ_2_ (**c**) ϕ_3_ and (**d**) ϕ_4_ tilts. Inset shows the tetrahedral ring in various orientations. Also shown are the atoms with respective labels. All the tilts exhibit anomalous behavior upon compression. At 0 GPa, the tilts compare well with previous experiments^37^. For the visualization of the tilts and their definition, please refer to the supplementary information (Supplementary Movies, Supplementary Figure S1).

The compressibility and elastic stiffness tensor of albite at high-pressure can be explained by the response of the alumino-silicate framework of tetrahedral units upon compression. In the albite crystal structure there are four distinct tetrahedral sites^26^,^35^, T_1o_, T_1m_, T_2o_ and T_2m_ (Fig. 3). The aluminum atoms occupy the T_1o_ site based on neutron diffraction^35^ and subsequent empirical and DFT studies indicated that the energetics of Al ordered in T_1o_ sites are of the order of 30 meV lower than T_2o_ sites^36^. In a recent study, it has been noted that variation in Al/Si order and exact chemistry of alkali site (Na or K) has only minor effect on the aggregate elasticity^21^. However, elasticity varies significantly across Na-Ca plagioclase series^22^. Upon compression, both the AlO_4_ and SiO_4_ tetrahedral units remain mostly rigid. The GGA and LDA predictions for the polyhedral moduli for the T_1o_ sites are, 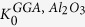 = 155.49 and 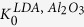 = 166.36 respectively. The GGA predictions for the polyhedral moduli for the T_1m_, T_2o_, and T_2m_ sites are, 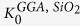 = 270.9, 229.3 and 257.4 whereas the LDA predictions for the polyhedral moduli for the T_1m_, T_2o_, and T_2m_ sites are 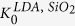 = 285.2, 204.3, 285.9 respectively. The pressure-induced compression in the aluminosilicate framework structure of albite is primarily accommodated by the gradual tilting and shearing of the cages formed by the tetrahedral units and is achieved by altering the T-O-T bond angles (Fig. 3; Supplementary Movies-1–4). In feldspar, four distinct tilts are recognized, i.e., ϕ_1_ to 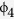 (Supplementary information). In triclinic albite, owing to lower symmetry, there are two distinct components for ϕ_1_ and ϕ_2_ tilts, i.e., ϕ_1_ is decomposed into ϕ_1o_ and ϕ_1m_. Similarly the tilt ϕ_2_ is decomposed into ϕ_2o_ and ϕ_2m_. In the present study all the tilt component shows anomalous behavior between 4–6 and 6–8 GPa for LDA and GGA respectively. This is a clear demonstration of the “cause and effect” relationship between the pressure dependent changes in the internal structure and the elasticity (Fig. 4). The flexibility of the alumino-silicate framework of tetrahedral unit results in significant anisotropy in elasticity. The a* direction in albite is significantly softer than the b* and c* direction.

**Figure 5:**
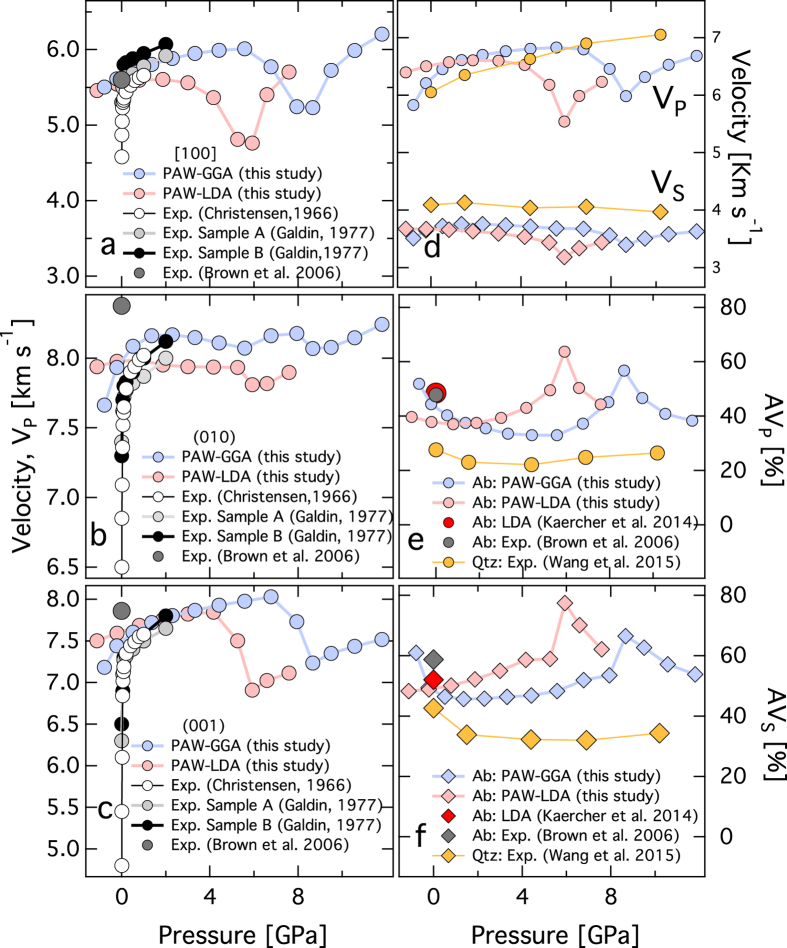
Seismic velocity and elastic anisotropy at high pressure. Compressional wave, V_P_ propagation directions (**a**) [100], (**b**)⊥ (010), (**c**)⊥ (001). LDA and GGA results are represented by filled red and blue symbols. Experimentally measured V_P_ as a function of pressure for albite (An5–8)^38^ (open white symbols) and oligoclase (An 10–30)^39^ (filled black symbols) to 1 and 2 GPa respectively. The recent experimental results^20^ are represented by filled grey symbol. The experimentally measured velocities^38^,^39^ are softer compared to the recent experiments^20^ for ⊥ (010) and ⊥ (001). (**d**) Bulk compressional wave V_P_ and shear V_S_ velocity as a function of pressure. For comparison, velocities for quartz^51^, a silicate framework mineral is also shown. (**e**) AV_P_, and (**f**) AV_S_ as a function of pressure. Also shown are the experimentally determined elastic anisotropy for albite^20^ and quartz^47^.

The pressure dependence of the P-wave velocity predicted from DFT is in very good agreement with previous measurements to pressures of 2.5 GPa^37^,^38^ (Fig. 5). Recent elasticity measurements predicts stiffer velocities for the b* and c* directions^19^ (Fig. 5). Although, the earlier work on feldspar megacrysts^37^,^38^ are “softer” relative to recent work^19^ in directions perpendicular to cleavage, i.e., b* and c* directions, the comparison is in better accord with the “true” single crystal behavior in the non-cleavage direction (100). It is likely that in the earlier experiments to determine the elastic constants^38^,^39^, the closing of the cracks at low-pressure lead to significantly greater pressure derivatives (Fig. 5). Agreement between all the previous experimental studies and present results are excellent for the softer a* direction. Substantial drop in compressional wave velocity, V_P_ occur between 4 and 8 GPa for the a* and c* crystallographic directions. These correspond to the discontinuous behavior of the individual elastic constants, *c*_11_, and *c*_33_ (Fig. 2). The pressure dependence for shear elastic constants *c*_44_ and *c*_66_ also low values between 4 and 8 GPa, which would correspond to velocity drops for V_S_ for propagation along a* and c* directions. Pressure-induced softening of the shear elastic constants have also been reported for the tetrahedraly coordinated silica (SiO_2_) polymorphs that shares corners and forms 3-D framework, as in quartz^40^,^41^,^42^,^43^,^44^ and coesite^45^,^46^.

The full elastic anisotropy compares quite well with recent experimental study^19^ and the DFT study^22^ (Fig. 5). The P-wave azimuthal anisotropy, AV_P_ decreases from 43% at 1 bar to 34% at 8 GPa to, whereas the S-wave anisotropy AV_S_ increases slightly from 46% at 1 bar to 49% at 8 GPa. At 1 bar, the anisotropy of P-wave to S-wave velocity ratios i.e., V_P_/V_S1_ and V_P_/V_S2_ are 68 and 59% respectively. Upon compression of albite to 8 GPa, the anisotropy in V_P_/V_S1_ and V_P_/V_S2_ reduces to 58 and 36% respectively.

The slight discrepancy elasticity and anisotropy between the previous DFT study and the present study is likely related to the differences in the computational parameters used. For instance, a higher energy cut-off of 800 eV is used in this study compared to 582 eV. In addition, the present study uses projector augmented wave method (Method section) as opposed to the pseudopotential method used in earlier DFT study^22^.

**Figure 6:**
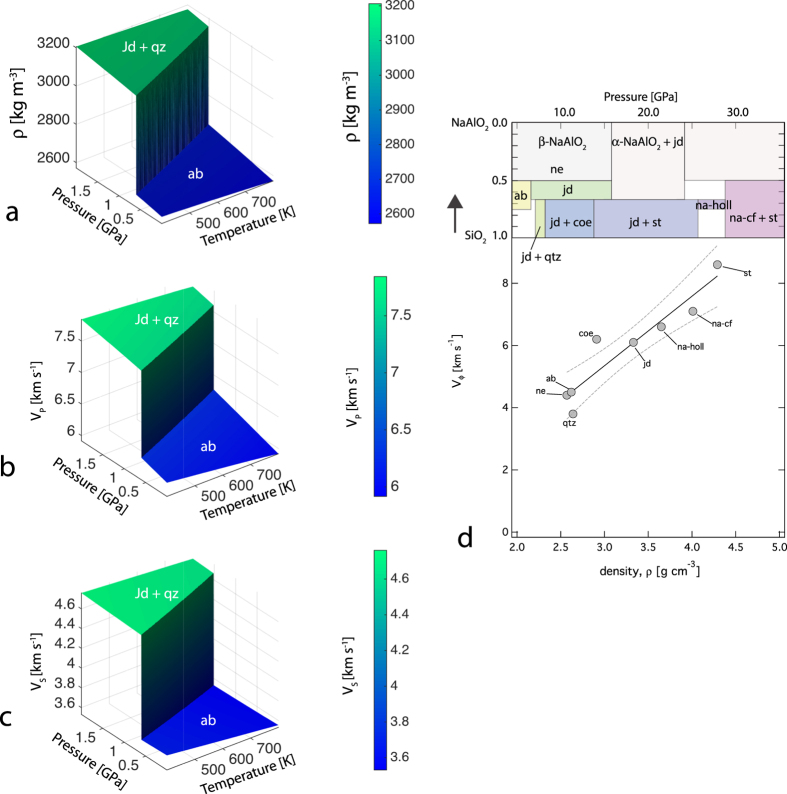
Albite transformation and geophysical discontinuity. (**a**) Plot of density (*ρ*), (**b**) Plot of compressional velocity (*V*_*P*_), and (**c**) Plot of shear wave velocity (*V*_*S*_) as a function of pressure and temperature across the decomposition of albite (ab) to a mixture of jadeite (jd) and quartz (qtz). (**d**) Plot of bulk sound velocity (*V*_*ϕ*_) for various mineral phases stable in SiO_2_-NaAlO_2_ join, modified from^53^. Mineral abbreviations- *qtz*, *coe,* and *st* refers to quartz, coesite, and stishovite with SiO_2_ stoichiometry i.e., silica polymorphs; *ab* and *na-holl* refers to albite and sodium hollandite with same stoichiometry NaAlSi_3_O_8_; *ne* and *na-cf* refers to nepheline and sodium calcium ferrite structured phase with NaAlSiO_4_ stoichiometry and *jd* refers to jadeite with NaAlSi_2_O_6_ stoichiometry. Density and bulk modulus data are derived from- *qtz*^47^; *coe*^54^; *st*^55^; *ab*^20^,^26^, this study; na-holl^56^; *ne*^57^, *na-cf*^58^,^59^.

The Mohorovicic discontinuity (Moho) marks the first major discontinuity separating the earth’s crust from the underlying denser mantle. The discontinuity is likely to be related to the changes in composition across crust and underlying mantle^48^,^49^. Across Moho the rock type is likely to change from gabbro consisting of pyroxene and plagioclase minerals to eclogite consisting of denser omphacite pyroxene and garnet. Several mineralogical transformations play important role in eclogitization and the proportion of mineral phase changes gradually across the transition rendering a continuous change in density and physical properties^49^. In a recent study, it has been suggested that for the thickened continental crust, the Moho could, in part, be explained by the transformation of albite (ab) to a mixture of jadeite (jd) and quartz (qtz)^50^. In this study, we used elasticity results of albite (ab) from this study and recent studies^19^,^26^ and combined them with the recent elasticity results of jadeite (jd)^51^,^52^ and quartz (qtz)^47^ to evaluate the discontinuity in compressional velocity across the univariant transformation: NaAlSi_3_O_8_ (*ab*) = NaAlSi_2_O_6_ (*jd*) + SiO_2_ (*qtz*). We used a thermodynamic code, Perple_X_^28^ and the thermodynamic database^27^ to predict the phase boundary and the changes in the physical properties such as density and P-, and S-wave velocity across the boundary (Fig. 6). In our analysis, the pressure derivatives of the velocities for albite i.e., 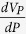 and 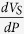 are from this study. The pressure derivatives of the velocities for jadeite^51^,^52^ and quartz^60^ are estimated from previous results. The temperature derivatives of the velocities i.e., 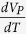 and 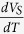 are derived from Perple_X_^28^. We find that at a depth of around ~40 km, the average P- and S-wave velocity of mineral assemblage consisting of jadeite and quartz is ~1.0 km/s greater than that of albite (Fig. 6). This is also very similar to the known contrast between crust and the mantle. The weighted average crustal P-wave velocity across several continental crustal settings is ~6.45 (±0.2) km/s where as the underlying average mantle velocity is ~8.09 (±0.2) km/s^61^ has a similar velocity contrast across the crust-mantle discontinuity.

Alkali oxide-Na_2_O is a minor component in the deep Earth mineralogical model^49^, metasomatism in subduction zones by Na-rich fluids could stabilize jadeite rich rocks- jadeitites and albite rich rocks- albitite which occur together with serpentinite melange^62^,^63^,^64^. In the deep Earth, beyond the thermodynamic stability field of albite and jadeite, alkalis including sodium could be incorporated into denser mineral phases such as Na- hollandite and calcium ferrite (*cf*) structured phase with NaAlSiO_4_ stoichiometry^53^. Upon compression, a series of mineral transformation and densification is likely to modify albite to jadeite + quartz/coesite/stishovite and eventually to Na-holandite^53^. Similarly, jadeite could also be transformed to a mixture of NaAlSiO_4_ (*cf* phase) and stishovite^57^. Both hollandite and *cf* phases have crystal structure consisting of SiO_6_ and AlO_6_ units that forms tunnels where alkali atom such as Na and K reside^49^,^65^,^66^,^67^. The density and elasticity of sodium bearing phases including albite exhibit a positive correlation. Recent experimental studies have indicated that sodium could also be incorporated in ringwoodite^68^. How sodium is partitioned between major mantle minerals i.e., magnesium silicates and minor aluminosilicate at high pressure remains unknown and will be important to constrain the fate of sodium in the deep Earth.

## Method

We investigated albite using density functional theory (DFT)^69^,^70^,^71^ [*Hohenberg and Kohn*, 1964; *Kohn and Sham*, 1965]. DFT-based studies of the energetics, equation-of state, and elasticity have been widely used to examine condensed matter including minerals that are stable in the Earth’s interior^75^,^76^,^77^,^78^,^79^,^80^. We used the local density approximation (LDA) and the semi-local generalized gradient approximation (GGA)^81^,^82^,^83^. In addition, we also used the projector augmented wave method (PAW)^84^ within the Vienna *ab initio* simulation package (VASP)^84^,^85^,^86^,^87^. We used an energy cut-off E_cut_ ranging from 400 eV to 1100 eV and *k*-point sampling ranging from 2 to 46 *k*-points in the irreducible wedge of the Brillouin zone. We find that the results are fully converged at an energy cut-off E_cut_ of 800 eV and a 3 × 2 × 3 *k*-point mesh Monkhorst-Pack^88^ (Supplementary Figure S2). The total energies and pressures were converged to within 1.06 meV and 0.35 GPa. We used the experimentally determined crystal structure of albite from X-ray and neutron diffraction^35^ as the input for the crystal structure and the starting point for a full structural optimization of the crystal structure. We performed all the calculations in the primitive unit cell (Space group: 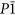, 52 atoms). The static (T = 0 K) calculation leads to primitive symmetry instead of the 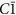 symmetry observed in experiments. For the determination of the elastic stiffness tensor, we strained the crystal structure and relaxed the internal degrees of freedom while preserving the symmetry of the crystal structure. The elastic constants *c*_*ijkl*_ were obtained by relating changes in stress with the applied strain, *σ*_*ij*_ = ∑_*kl*_ *c*_*ijkl*_*ε*_*kl*_. We applied ±1% strains to accurately determine the stresses in the limit of small strain (*ε*_*kl*_ → 0)^29^,^34^. The *Cartesian* frame for the elastic constants has X_2_ parallel to crystallographic axis *b*, X_3_ parallel to c*, and X_1_ normal to X_2_ and X_3_, forming a right-handed system for triclinic symmetry. We used the petrophysical software to determine the elastic anisotropy^89^. The P-wave and S-wave anisotropy are defined as 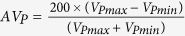 and 
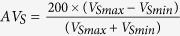
 respectively.

## Additional Information

**How to cite this article**: Mookherjee, M. *et al*. Pressure induced elastic softening in framework aluminosilicate- albite (NaAlSi_3_O_8_). *Sci. Rep.* **6**, 34815; doi: 10.1038/srep34815 (2016).

## Supplementary Material

Supplementary Movie S1

Supplementary Movie S2

Supplementary Movie S3

Supplementary Movie S4

Supplementary Information
