# Supplementary material for: Pressure induced elastic softening in framework aluminosilicate- albite (NaAlSi3O8)
Source: Sci Rep. 2016 Oct 13;6:34815. doi: 10.1038/srep34815 (PMC5062091; doi:10.1038/srep34815)
Supplement: Supplementary Information [file srep34815-s5.pdf]

Supporting Information for

# Pressure induced elastic softening in framework aluminosilicate- albite ($NaAlSi_3O_8$)

Mainak Mookherjee[a*], David Mainprice[b], Ketan Maheshwari[c], Olle Heinonen[d], Dhenu Patel[a], Anant Hariharan[e]

[a]Earth, Ocean and Atmospheric Sciences, Florida State University, Tallahassee, FL, 32310, USA.

[*]corresponding author's email: mmookherjee@fsu.edu

[b]Géosciences Montpellier UMR CNRS 5243, Université Montpellier II, 34095, Montpellier, France.

[c]Center of Simulation and Modeling, University of Pittsburgh, Pittsburgh, PA, 15260.

[d]Materials Science Division, Argonne National Laboratory, Argonne, IL 60439, USA.

[e]Earth and Atmospheric Sciences, Cornell University, NY 14850, USA.

## Tetrahedral tilts for framework silicate:

The three dark blue tetrahedral represents $SiO_4$ units, whereas the light-blue unit represents the $AlO_4$ unit. The compression is accommodated in framework structures by tilting of the tetrahedral units[1,2]. There are four distinct tilts $\phi_1$ to $\phi_4$. For the labeling of the atoms, please refer to the inset of **Figure 4** in the main text.

**Supplementary Movie: Mookherjee_Suppl.Info_Albite_Tilt1|**

The movie shows the $\phi_1$ tilt -characterized by the tilt of the T1o and T1m tetrahedral units. Ideally the tetrahedral-base of T1o and T1m should be parallel to the a-c plane. The $\phi_1$ tile captures the deviation, i.e., the angle formed by the tetrahedral basal plane from the a-c plane. Modifying Megaw's[2] original definition for the ideal tetrahedral ring, the

triclinic albite will have two distinct $\phi_1$ tilts, we also incorporate the atomic labels used in the present study (**Figure 3, 4**), the tilt of the T1o is defined as $\phi_{1o} = \sin^{-1}\left[\frac{b(y_{O_{a1}} - y_M)}{\left|O_{a1} - O_M\right|}\right]$ where $O_M$ is the hypothetical, mid-point between Obo and Odo and hence $y_M = \frac{1}{2}(y_{bo} + y_{do})$. Similarly, the tilt of the T1m is defined as $\phi_{1m} = \sin^{-1}\left[\frac{b(y_{O_{a1}} - y_{M'})}{\left|O_{a1} - O_{M'}\right|}\right]$ where $O_{M'}$ is the hypothetical, mid-point between Obm and Odm and hence $y_M = \frac{1}{2}(y_{bm} + y_{dm})$.

**Supplementary Movie: Mookherjee_Suppl.Info_Albite_Tilt2, 3|**

The movie shows the $\phi_2$ tilt -characterized by the tilt of the T2m and T2o tetrahedral units. For the T2o tetrahedral unit, if we define the hypothetical mid-point of the tetrahedral edge Ocm-Oa2 as $O_P$ and the hypothetical mid-point of the tetrahedral edge Odm-Obo as $O_N$, the $\phi_{2o}$ tilt is defined as the deviation of the PN vector from the ***a-c*** plane, i.e., $\phi_{2o} = \sin^{-1}\left[\frac{b(y_N - y_P)}{\left|O_P - O_N\right|}\right]$, where $y_P = \frac{1}{2}(y_{cm} + y_{a2})$ and $y_N = \frac{1}{2}(y_{dm} + y_{bo})$ and OP-ON is the distance between the mid-points P and N., Similarly for the T2m tetrahedral unit hypothetical mid-point of the tetrahedral edge Oco-Oa2 as $O_{P'}$ and the hypothetical mid-point of the tetrahedral edge Odo-Obm as $O_{N'}$. And the tilt is $\phi_{2m}$ characterized by $\phi_{2m} = \sin^{-1}\left[\frac{b(y_{N'} - y_{P'})}{\left|O_{P'} - O_{N'}\right|}\right]$ where $y_{P'} = \frac{1}{2}(y_{co} + y_{a2})$ and $y_{N'} = \frac{1}{2}(y_{do} + y_{bm})$ and OP'-ON' is the distance between the mid-points P' and N'.

The tetrahedral tilt $\phi_3$ is defined as $\phi_3 = \sin^{-1}\left[\frac{b(y_{bo} - y_{dm})}{|O_L - O_{bo}|}\right]$ where, OL is the hypothetical midpoint between Obo-Odo edge. The movie shows both $\phi_2$ and $\phi_3$ tilts as a function of pressure.

**Supplementary Movie: Mookherjee_Suppl.Info_Albite_Tilt2, 3a|**

The movie shows the same as **Mookherjee_Suppl.Info_Albite_Tilt2, 3,** however, additional tetrahedral units, the cage that hosts the Na atoms and the outline of the unit cell is also shown.

**Supplementary Movie: Mookherjee_Suppl.Info_Albite_Tilt4|**

Albite crystal structure is viewed down the ***b***- axis. The rings formed by four tetrahedral units are shown. The $\phi_4$ tilt is best viewed down the ***b***- axis. The four tetrahedral units form a ring, with the bridging oxygen atoms Odo, Obo, Odm and Obm. The movie shows the coordinated movement of Odo-Odm inwards and Obo-Obm outwards causes shearing of the ring. The shearing tilt is characterized by the formula, $\phi_4 = \frac{1}{4}\sum_{i=1,4}\left|angle_i(OOO) - 90^o\right|^1$, where 90° is the ideal angle formed by Obm-Odo-Obo, Odo-Obo-Odm, Obo-Odm-Obm, and Odm-Obm-Odo. The variation of the individual angles is shown in **Supplementary Figure 1**. The comparison with experimental results is quite well.

**Table Captions**

**Table S1|** Equation of state parameters, $V_0$ , $K_0$ , and $K_0'$ for albite ($NaAlSi_3O_8$)

*Table S1 footnote*: *equation of state parameters are for analbite ($NaAlSi_3O_8$) but with complete disorder of Al, Si in the tetrahedral sites[3]

**Table S2|** Unit-cell volume, density ($\rho$), full elastic constant tensor ($c_{ij}$), Hill averages of bulk ($K_{Hill}$) and shear ($G_{Hill}$) moduli of triclinic albite as a function of pressure. Predicted elasticity results from DFT simulations (**a**) PAW-GGA and (**b**) PAW-LDA.

## Supplementary Figure Captions

**Supplementary Figure 1|** Shows the plot of O-O-O angles as a function of pressure. The figures are labeled with the hinge oxygen atoms, i.e., (upper left) Obm, (middle left) Obo, (lower left) Oco, (upper right) Odm, (middle-right) Odo , and (lower right) Ocm. The red and blue symbols represent results from PAW-LDA and PAW-GGA (this study), the white[4] and grey[5] symbols are from experiments.

**Supplementary Figure 2|** The left panel shows the plot of Total energy vs. Energy Cut-off. It clearly shows that at energy cut-off greater than 800 eV the total energy does not decrease significantly. The right panel shows the convergence test for the total energy as a function of # of irreducible k-points. The total energy corresponding to a Monkhorst mesh of 3x2x3 is converged. Also shown is the time required for computation.

**References and Notes**


1. Angel, R. J., Sochalski-Kolbus, M., Tribaudino, M. 2012. Tilts and tetrahedral: The origin of the anisotropy of feldspars, Am. Mineral., 97, 765-778.
2. Megaw, H. D. 1974. Tilts and tetrahedral in feldspars. In W.S. Mckenzie and J. Zussman, Eds. The Feldspars, 87-113. Manchester United Press, Crane, Russak & Co.
3. Curetti, N., Schhalski-Kolbus, L. M., Angel, R. J., Benna, P., Nestola, F., Bruno, E. 2011. High-pressure structural evolution and equation of state of analbite. Am. Mineral., 96, 383-392.
4. Downs, R. T., Hazen, R. M., Finger, L. W., 1994. The high-pressure crystal chemistry of low albite and the origin of the pressure dependency of Al-Si ordering. Am. Mineral., 79, 1042-1052.
5. Benusa, M. D., Angel, R. J., Ross, N. L., 2005. Compression of albite, $NaAlSi_3O_8$. Am. Mineral., 90, 1115-1120.

**Table S1|**

| $V_0$ (Å³) | $K_0$ (GPa) | $K_0'$ | Method | Ref. |
|---|---|---|---|---|
| 664.39 (0.12) | 69.9 (n.d.) | - | SCXRD | Angel *et al*. 1988 |
| 664.04 (0.09) | 54.0 (1.0) | 6.0 (1.0) | SCXRD | Downs *et al*. 1994 |
| 664.04 (0.09) | 53.8 (0.9) | 6.0 (0.6) | SCXRD | Angel 2003 |
| 664.76 (0.85) | 52.3 (0.9) | 8.8 (0.6) | SCXRD | Benusa *et al*. 2005 |
| 665.87 (0.04) | 56.4 (0.7) | 3.9 (0.3) | SCXRD | Tenner *et al*. 2007 |
| 667.04 (0.50) | 50.3 (0.5) | 8.9 (0.5) | SCXRD | Curetti *et al*. 2011[3]* |
| 687.40 (0.30) | 51.7 (0.9) | 4.7 (0.3) | DFT | PAW-GGA (this study) |
| 637.90 (0.20) | 59.9 (0.4) | 4.6 (0.4) | DFT | PAW-LDA (this study) |

**Table S2a.**

| | GGA | | | | | | | | | | | | | | *finite strain fit* | |
|---|---|---|---|---|---|---|---|---|---|---|---|---|---|---|---|---|
| V [Å$^3$] | 700.0 | 690.0 | 680.0 | 670.0 | 660.0 | 650.0 | 640.0 | 630.0 | 620.0 | 610.0 | 600.0 | 590.0 | 580.0 | 570.0 | 687.4 | |
| ρ [g/cm$^3$] | 2.49 | 2.52 | 2.56 | 2.60 | 2.64 | 2.68 | 2.72 | 2.76 | 2.81 | 2.85 | 2.90 | 2.95 | 3.00 | 3.05 | 2.53 | |
| P [GPa] | -0.78 | -0.22 | 0.51 | 1.36 | 2.30 | 3.32 | 4.42 | 5.58 | 6.78 | 7.94 | 8.67 | 9.51 | 10.57 | 11.81 | 0.00 | |
| $c_{11}$ | 73.28 | 77.53 | 81.97 | 86.35 | 90.22 | 93.80 | 96.64 | 98.70 | 92.42 | 77.77 | 77.67 | 95.54 | 106.53 | 116.31 | 78.84 | 6.11 |
| $c_{22}$ | 146.19 | 158.54 | 167.26 | 173.04 | 176.04 | 177.82 | 178.74 | 179.71 | 187.12 | 190.43 | 188.07 | 191.46 | 198.21 | 206.46 | 160.95 | 11.20 |
| $c_{33}$ | 122.41 | 134.69 | 143.85 | 151.39 | 157.47 | 162.75 | 168.09 | 172.84 | 178.82 | 168.20 | 148.79 | 156.30 | 162.59 | 168.90 | 136.75 | 11.31 |
| $c_{12}$ | 22.79 | 28.67 | 33.92 | 38.11 | 41.65 | 44.62 | 47.08 | 49.55 | 49.73 | 47.19 | 40.74 | 47.01 | 53.88 | 60.42 | 29.91 | 6.44 |
| $c_{13}$ | 24.79 | 31.02 | 36.43 | 41.28 | 45.79 | 50.39 | 54.71 | 58.54 | 57.32 | 42.70 | 29.20 | 37.82 | 42.59 | 46.39 | 32.47 | 6.69 |
| $c_{23}$ | -4.23 | 5.63 | 12.82 | 18.18 | 22.34 | 26.15 | 29.72 | 32.51 | 37.40 | 38.00 | 28.90 | 31.19 | 35.17 | 39.44 | 5.63 | 9.48 |
| $c_{14}$ | 2.73 | 2.79 | 2.80 | 2.78 | 3.16 | 3.47 | 3.77 | 4.08 | 3.73 | 6.32 | 5.65 | 2.41 | 0.93 | 0.09 | 2.82 | 0.09 |
| $c_{15}$ | -9.41 | -8.77 | -7.95 | -7.35 | -7.01 | -6.77 | -6.74 | -6.73 | -6.10 | -4.69 | -6.37 | -7.41 | -8.00 | -9.02 | -8.55 | 0.97 |
| $c_{16}$ | -2.29 | -2.53 | -2.73 | -3.04 | -3.34 | -3.53 | -3.49 | -3.80 | -4.78 | 0.45 | 7.13 | 4.96 | 3.62 | 2.95 | -2.59 | -0.21 |
| $c_{24}$ | -1.41 | -2.99 | -4.06 | -4.23 | -4.06 | -3.77 | -3.23 | -2.73 | -1.74 | 0.19 | 3.67 | 4.42 | 4.79 | 5.25 | -3.16 | -0.92 |
| $c_{25}$ | -2.76 | -0.23 | 1.91 | 3.37 | 4.22 | 4.80 | 5.06 | 5.20 | 5.74 | 5.99 | 5.59 | 5.34 | 5.59 | 5.76 | 0.04 | 2.56 |
| $c_{26}$ | 1.12 | 0.51 | 0.11 | -0.07 | 0.26 | 0.92 | 2.01 | 3.15 | 2.60 | 4.50 | 10.97 | 12.27 | 11.74 | 10.33 | 0.39 | -0.49 |
| $c_{34}$ | -1.46 | -2.99 | -4.13 | -4.31 | -3.63 | -2.95 | -2.11 | -1.25 | -0.48 | 3.26 | 4.45 | 0.79 | -1.38 | -2.48 | -3.20 | -1.13 |
| $c_{35}$ | -24.31 | -22.63 | -21.20 | -20.30 | -20.02 | -19.69 | -19.54 | -19.25 | -18.26 | -16.35 | -17.50 | -18.99 | -19.85 | -20.74 | -22.59 | 1.71 |
| $c_{36}$ | -1.86 | -2.99 | -3.72 | -4.06 | -4.23 | -4.29 | -4.14 | -3.67 | -3.23 | 1.67 | 11.29 | 10.95 | 10.44 | 10.52 | -3.13 | -0.83 |
| $c_{45}$ | 8.22 | 6.10 | 4.42 | 3.34 | 2.52 | 1.92 | 1.49 | 1.27 | 1.67 | 1.85 | 3.39 | 3.88 | 4.22 | 4.89 | 5.75 | -1.87 |
| $c_{46}$ | -1.08 | -0.73 | -0.58 | -0.61 | -0.51 | -0.47 | -0.31 | -0.17 | -1.58 | -3.50 | -4.63 | -1.40 | 1.42 | 3.89 | -0.69 | 0.09 |
| $c_{56}$ | 3.23 | 2.99 | 3.06 | 3.30 | 3.49 | 3.92 | 4.19 | 4.65 | 6.08 | 6.19 | 6.85 | 6.47 | 5.57 | 4.78 | 3.05 | 0.15 |
| $c_{44}$ | 16.59 | 19.72 | 22.31 | 24.19 | 25.46 | 26.36 | 27.23 | 27.96 | 29.54 | 29.10 | 29.41 | 31.63 | 33.76 | 36.01 | 20.35 | 2.94 |
| $c_{55}$ | 29.28 | 31.37 | 33.51 | 35.30 | 36.37 | 37.41 | 38.28 | 39.01 | 40.71 | 41.33 | 42.10 | 42.31 | 42.15 | 41.77 | 31.92 | 2.59 |
| $c_{66}$ | 37.21 | 37.30 | 37.15 | 36.56 | 35.55 | 34.38 | 33.24 | 31.86 | 30.96 | 26.71 | 19.69 | 21.40 | 24.25 | 26.69 | 37.83 | -1.08 |
| $K_{Hill}$ | 43.64 | 52.49 | 59.24 | 64.56 | 68.82 | 72.66 | 76.01 | 78.87 | 79.21 | 70.71 | 59.14 | 69.24 | 76.50 | 82.75 | 53.87 | 4.70 |
| $G_{Hill}$ | 30.63 | 33.61 | 35.50 | 36.68 | 37.18 | 37.41 | 37.53 | 37.46 | 37.91 | 36.23 | 33.52 | 36.30 | 38.53 | 40.24 | 34.74 | 2.81 |

**Table S2b.**

| | LDA | | | | | | | | | | *finite strain fit* | |
|---|---|---|---|---|---|---|---|---|---|---|---|---|
| V [Å$^3$] | 650.0 | 640.0 | 630.0 | 620.0 | 610.0 | 600.0 | 590.0 | 580.0 | 570.0 | 560.0 | 637.9 | |
| ρ [g/cm$^3$] | -1.12 | -0.21 | 0.80 | 1.87 | 3.00 | 4.16 | 5.26 | 5.93 | 6.59 | 7.59 | 2.73 | |
| P [GPa] | 2.68 | 2.72 | 2.76 | 2.81 | 2.85 | 2.90 | 2.95 | 3.00 | 3.05 | 3.11 | 0.00 | |
| $c_{11}$ | 78.71 | 82.57 | 85.37 | 87.23 | 86.90 | 82.00 | 67.72 | 66.62 | 88.22 | 100.32 | 83.30 | 3.20 |
| $c_{22}$ | 168.79 | 173.02 | 176.06 | 177.61 | 179.58 | 182.62 | 185.55 | 181.94 | 185.02 | 192.76 | 173.12 | 3.30 |
| $c_{33}$ | 146.31 | 152.72 | 159.33 | 165.53 | 171.25 | 175.62 | 163.48 | 139.61 | 146.82 | 153.52 | 154.40 | 6.84 |
| $c_{12}$ | 36.64 | 40.57 | 43.38 | 45.11 | 46.44 | 47.33 | 43.83 | 36.58 | 45.08 | 52.42 | 40.79 | 2.89 |
| $c_{13}$ | 40.10 | 45.04 | 49.63 | 53.94 | 56.94 | 56.74 | 40.85 | 24.26 | 33.66 | 37.81 | 46.26 | 5.07 |
| $c_{23}$ | 16.63 | 21.34 | 25.58 | 29.36 | 32.90 | 36.80 | 37.31 | 26.44 | 29.33 | 33.67 | 21.83 | 4.45 |
| $c_{14}$ | 3.53 | 3.71 | 3.79 | 3.82 | 3.65 | 3.72 | 5.79 | 5.92 | 2.64 | 1.40 | 3.72 | 0.04 |
| $c_{15}$ | -5.92 | -5.45 | -5.12 | -5.04 | -4.96 | -4.57 | -3.44 | -4.91 | -5.79 | -6.82 | -5.57 | 0.28 |
| $c_{16}$ | -3.57 | -4.09 | -4.59 | -5.19 | -5.97 | -6.50 | -1.57 | 6.66 | 4.57 | 3.64 | -4.19 | -0.59 |
| $c_{24}$ | -3.84 | -3.52 | -3.01 | -2.38 | -1.59 | -0.71 | 1.16 | 5.01 | 5.58 | 5.99 | -3.39 | 0.61 |
| $c_{25}$ | 3.34 | 4.49 | 5.29 | 5.66 | 5.94 | 5.99 | 6.34 | 5.64 | 5.17 | 5.49 | 4.60 | 0.95 |
| $c_{26}$ | -0.34 | -0.36 | 0.01 | 0.82 | 1.59 | 2.41 | 4.30 | 12.25 | 14.08 | 13.05 | -0.35 | 0.01 |
| $c_{34}$ | -3.84 | -3.54 | -2.89 | -2.12 | -1.16 | 0.20 | 3.81 | 5.61 | 0.81 | -1.45 | -3.38 | 0.50 |
| $c_{35}$ | -22.48 | -21.91 | -21.49 | -21.16 | -20.72 | -19.89 | -17.53 | -18.37 | -20.18 | -21.17 | -21.93 | 0.48 |
| $c_{36}$ | -4.28 | -4.41 | -4.35 | -4.03 | -3.71 | -3.34 | 1.06 | 12.12 | 11.53 | 11.07 | -4.30 | 0.01 |
| $c_{45}$ | 2.56 | 1.77 | 1.21 | 0.86 | 0.79 | 0.96 | 1.43 | 3.35 | 4.25 | 4.98 | 1.65 | -0.72 |
| $c_{46}$ | -0.37 | -0.48 | -0.65 | -0.90 | -1.16 | -1.68 | -3.54 | -4.99 | -0.70 | 2.63 | -0.50 | -0.23 |
| $c_{56}$ | 3.84 | 4.22 | 4.65 | 5.25 | 5.25 | 6.74 | 6.88 | 7.76 | 7.58 | 6.56 | 4.27 | 0.42 |
| $c_{44}$ | 26.84 | 28.16 | 29.34 | 30.20 | 30.94 | 31.47 | 31.07 | 30.87 | 33.00 | 35.15 | 28.34 | 1.20 |
| $c_{55}$ | 34.01 | 35.56 | 36.76 | 37.89 | 38.81 | 39.64 | 40.43 | 40.69 | 39.80 | 39.11 | 35.69 | 1.39 |
| $c_{66}$ | 36.42 | 35.36 | 33.98 | 32.38 | 30.71 | 28.82 | 24.77 | 16.48 | 19.48 | 23.21 | 35.03 | -1.47 |
| $K_{Hill}$ | 61.39 | 66.10 | 70.02 | 73.11 | 75.18 | 75.21 | 66.19 | 51.57 | 63.98 | 71.67 | 66.83 | 4.60 |
| $G_{Hill}$ | 36.15 | 36.70 | 37.00 | 37.04 | 36.85 | 36.29 | 34.86 | 30.45 | 34.06 | 36.78 | 36.70 | 0.35 |

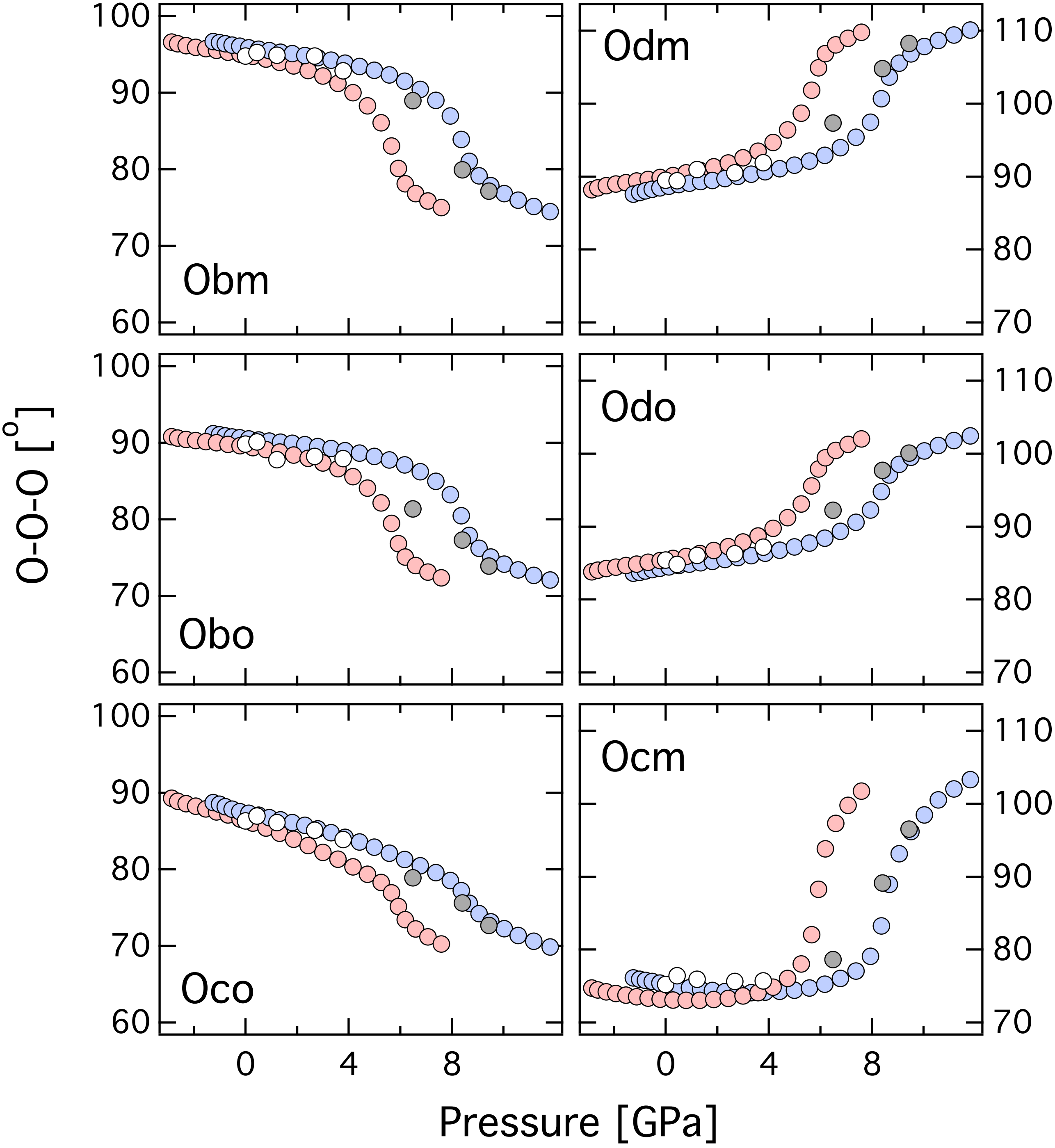

Obm
Odm
Obo
Odo
Oco
Ocm
O-O-O [°]
O-O-O [°]
Pressure [GPa]
100
90
80
70
60
110
100
90
80
70
0
4
8

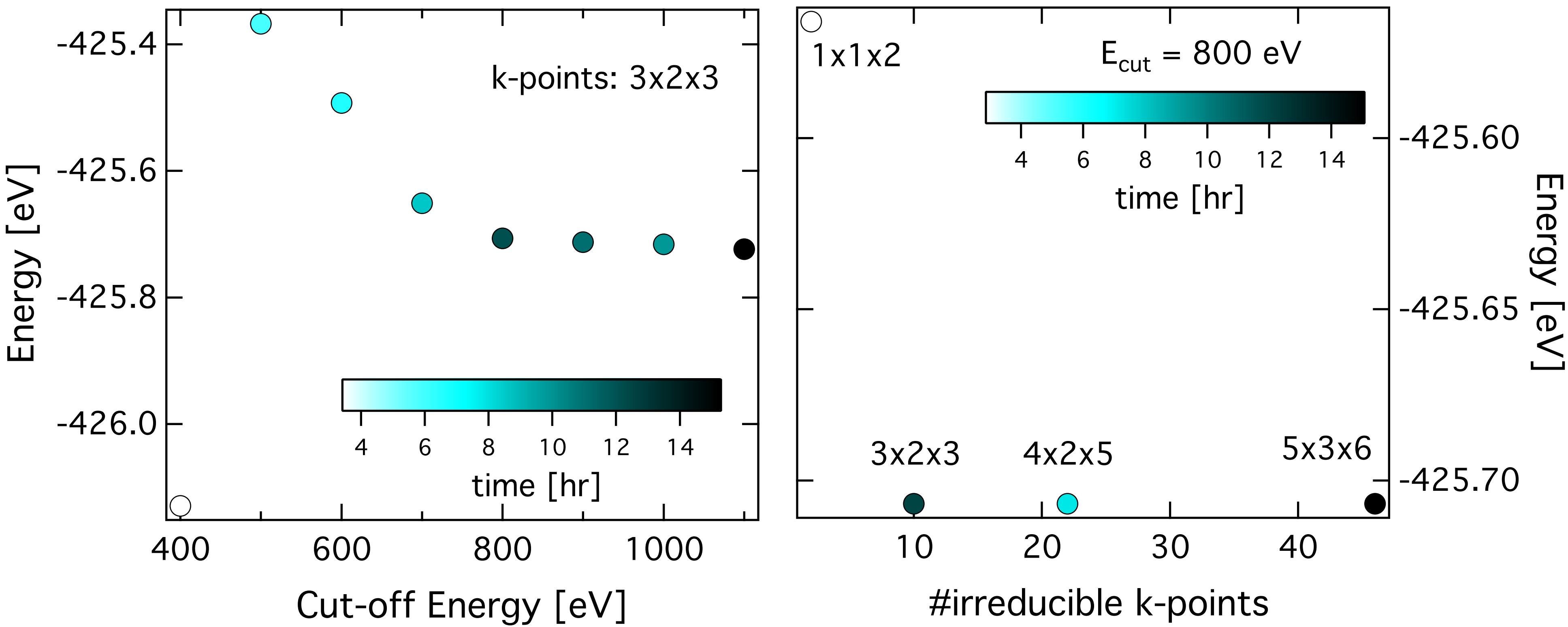

Energy [eV]
-425.4
-425.6
-425.8
-426.0
k-points: 3x2x3
4
6
8
10
12
14
time [hr]
400
600
800
1000
Cut-off Energy [eV]
1x1x2
$E_{cut}$ = 800 eV
4
6
8
10
12
14
time [hr]
3x2x3
4x2x5
5x3x6
10
20
30
40
#irreducible k-points
-425.60
-425.65
-425.70
Energy [eV]
